# Implantation depth and its influence on complications after TAVI with self-expanding valves

**DOI:** 10.1007/s10554-021-02275-3

**Published:** 2021-05-14

**Authors:** Philipp Breitbart, Jan Minners, Manuel Hein, Holger Schröfel, Franz-Josef Neumann, Philipp Ruile

**Affiliations:** 1 Division of Cardiology & Angiology II, University Heart Center Freiburg-Bad Krozingen, Südring 15, 79189 Bad Krozingen, Germany; 2 Division of Cardiovascular Surgery, University Heart Center Freiburg-Bad Krozingen, Bad Krozingen, Germany

**Keywords:** TAVI, Computed tomography angiography, Fusion imaging, THV positioning, TAVI complications, Self-expanding valve types

## Abstract

Prior studies in patients with transcatheter aortic valve implantation (TAVI) demonstrated an influence of transcatheter heart valve (THV) position on the occurrence of new conductions disturbances (CD) and paravalvular leakage (PVL) post TAVI in balloon-expandable valves (BEV). Purpose of this study was to investigate the THV implantation depth and its influence on the occurrence of CD and PVL in self-expanding valves (SEV). We performed fusion imaging of pre- and post-procedural computed tomography angiography in 104 TAVI-patients (all with Evolut R) to receive a 3-D reconstruction of the THV within the native annulus region. The THV length below the native annulus was measured for assessment of implantation depth. Electrocardiograms pre-discharge were assessed for conduction disturbances (CD), PVL was determined in transthoracic echocardiography. The mean implantation depth of the THV in the whole cohort was 4.3 ± 3.0 mm. Using the best cut-off of ≥ 4 mm in receiver operating characteristic curve analysis (sensitivity 83.3%, specificity 60.0%) patients with lower THV position developed more new CD after TAVI (68.2 vs. 23.7%, P < 0.001). A deep THV position was identified as the only predictor for new CD after TAVI (odds ratio [CI] 1.312[1.119–1.539], P = 0.001). The implantation depth showed no influence on the grade of PVL (r = 0.052, P = 0.598). In patients with TAVI using the Evolut R SEV, a lower THV positioning (≥ 4 mm length below annulus) was a predictor for new conduction disturbances. In contrast, implantation depth was not associated with the extent of PVL.

**Graphic abstract:**

Prostheses positions of self-expanding valves and their influence on the occurrence of new conduction disturbances and the grade of paravalvular leakage after TAVI.
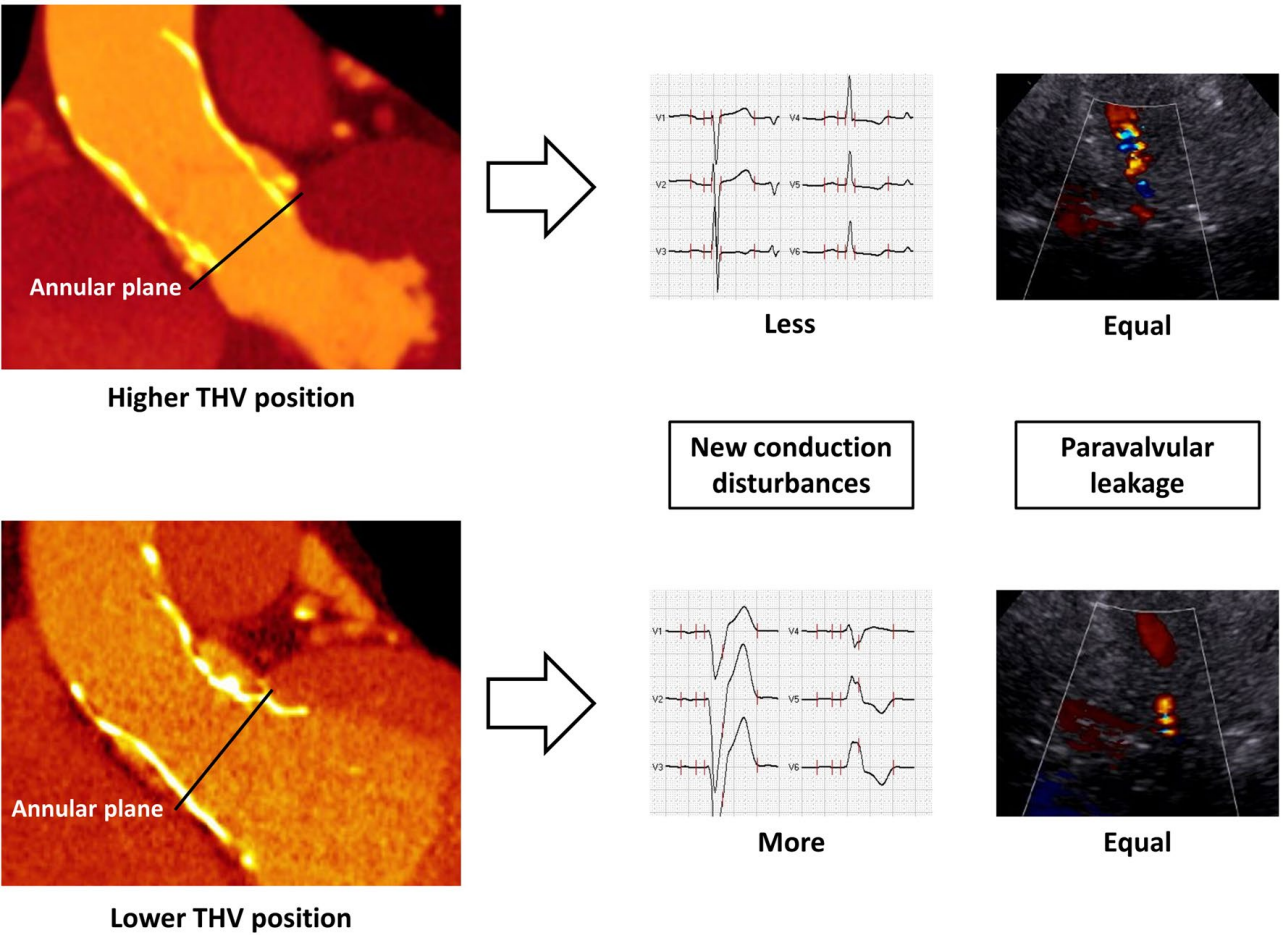

## Introduction

Transcatheter aortic valve implantation (TAVI) is an established treatment alternative to surgical aortic valve replacement in symptomatic aortic stenosis of patients with intermediate to high surgical risk [1]. The most common prosthetic valve types are self-expanding valves (SEV) and balloon-expandable valves (BEV). A large randomized study demonstrated equivalence for both systems with regard to valve-related efficacy endpoints [2]. Yet, a recent study revealed significantly higher rates of paravalvular leakage (PVL) and permanent pacemaker implantation caused by new-onset conduction disturbances (CD) in patients with SEV [3]. These factors are known to be transcatheter heart valve (THV) position related [4–6]. A new method of fusion imaging of pre- and post-procedural computed tomography angiography (CTA), published by our group, facilitates a three dimensional visualization of the THV within the native annulus plane after TAVI [7]. Using this method, we revealed a deep implantation of the THV as predictor for new-onset conduction disturbances in patients with BEV [7].

Therefore, we aimed to investigate the THV position of self-expanding valves assessed by fusion imaging method of pre- and post-TAVI CTA and its influence on the occurrence of new CD and PVL.

## Methods

### Study population

In accordance to the guidelines after thoracic aortic stent implantation a post-TAVI CTA was performed in all patients within our institution [8]. The purpose of this CTA was to identify possible complications, e.g. aortic injuries or thrombosis of the valves. Reasons for not performing a post-procedural CTA were described previously, e.g. renal insufficiency or frailty [9]. All patients with evaluable pre- and post-TAVI CTA and implanted newer generation SEV (Evolut R, Medtronic Inc., Minneapolis, USA) between January 2015 and June 2020 were candidates for study inclusion. Patients with valve-in-valve procedures were excluded. Experienced operators (each with an experience of at least 100 TAVI-procedures) implanted all THVs via a transfemoral access. The multidisciplinary, institutional heart team decided on TAVI eligibility, procedural feasibility as well as the preferred access route or prosthesis type and size [10]. All patients gave written informed consent for TAVI and the anonymized use of clinical, procedural, and follow-up data at the time of the intervention. The study was approved by the local institutional review board and complies with the Declaration of Helsinki.

### Image acquisition

Our detailed CTA-protocol was described previously [9]. In brief, we used a second generation dual-source CT scanner (Somatom Definition Flash, Siemens Healthcare, Forchheim, Germany) for the retrospective ECG-gated contrast-enhanced pre- and post-TAVI CTAs (70 mL for pre- and 50 mL for post-TAVI CTA, Imeron 400, Bracco, Konstanz, Germany) [9]. The post-TAVI CTAs were mostly performed between the second and seventh day after the intervention.

We used the “bolus tracking”-technique for beginning the CTA-scans with a region of interest in the left atrium. Images were reconstructed at 50 ms steps throughout the cardiac cycle. The image analysis was conducted by two experienced readers in consensus (P.B. and P.R.) using a post-processing workstation (Syngo Multimodality Workplace, Siemens Healthcare, Forchheim, Germany).

### Image analysis

We carried out the measurements of the aortic annulus inclusive the area derived diameter and annulus eccentricity during systole on the pre-TAVI images. Additionally, these sequences were used for a calcification assessment of the device-landing zone. The degree of calcification was visually quantified for each cusp: grade 0: no calcification, grade 1: mild calcification as small calcified spots with minimal diameter ≤ 2 mm, grade 2: moderate calcification as calcified spots with minimal diameter more than 2 mm, grade 3: severe calcification as large calcified formations more than 5 mm minimal diameter [11].

**Fig. 1:**
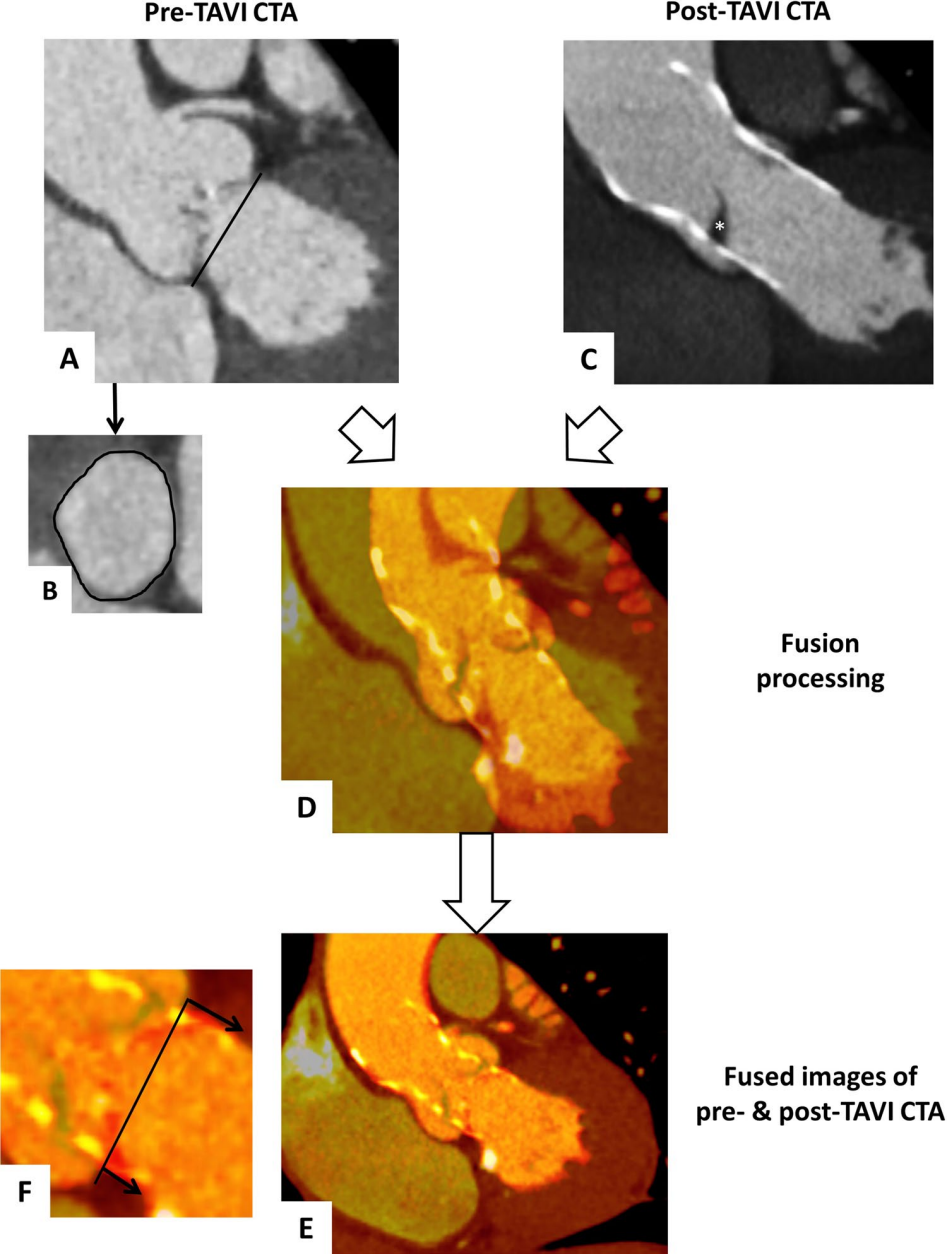
Visualization of fusion imaging process. Pre-TAVI CTA sagittal oblique (**a**) and axial (**b**) reconstructions with delineated annulus plane and post-TAVI CTA sagittal oblique reconstruction with the implanted Evolut R (**c**, * marked a leaflet thrombosis***)***. After semi-automatically merging of the pre- and post-TAVI CTAs (**d**), we manually adapted the fused images for an optimal alignment of the device-landing zone (**e**). Finally, the THV distances below the native annulus were measured next to all three cusps to assess the prosthesis implantation depth (**f**, *arrows for distance measurements****)***.—marked the annulus plane

As previously described by our group, fusion imaging of pre- and post-procedural CTA was used for an assessment of the final prosthesis position [7] (Fig. 1). To assess the implantation depth, we measured the THV distance below the native annulus [separately for left coronary cusp (LCC), right coronary cusp (RCC) and non-coronary cusp (NCC)] within the fusion images.

**Fig. 2:**
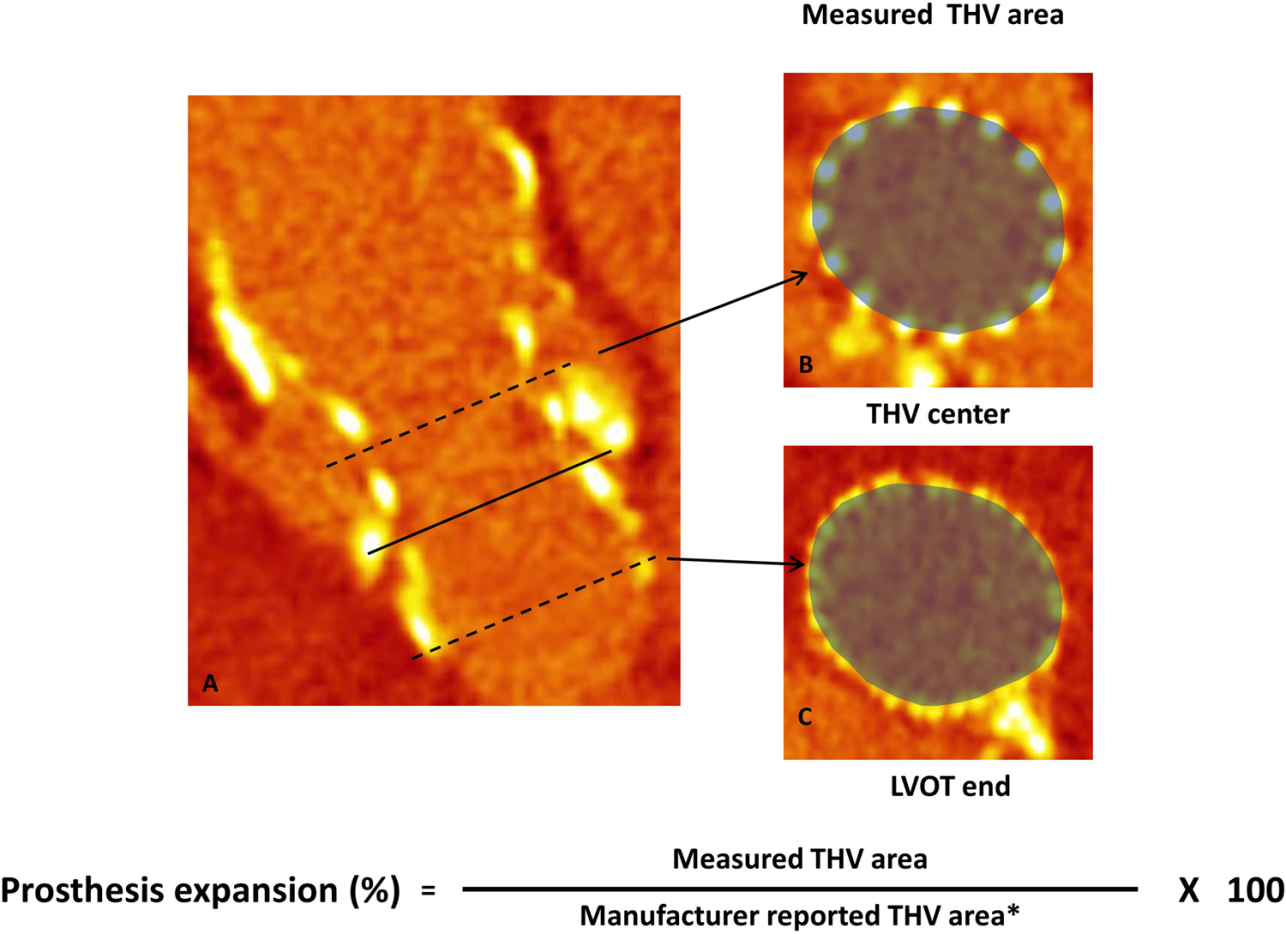
Determination of prosthesis expansion. Pre-TAVI CTA sagittal oblique (**a**, — marked the annulus plane, - - marked the heights of THV area measurements***)*** and axial (**b**,**c**) reconstructions for measurement of the prosthesis area on the height of the THV center (**b**) and at the LVOT end (**c**). The measured values are set in relation to the manufacturer reported ones to determine the prosthesis expansion

We determined THV tilt in relation to the annulus plane as the arctangent of (maximum − minimum stent center height above the annulus plane adjacent the individual cusps)/mean expanded THV diameter * 180/π) [11]. The THV area measurements of the stent center and the left ventricular outflow tract end (LVOT) were used to calculate the prosthesis expansion differentiated for both heights as (measured THV area/manufacturer reported THV area for the respective height) × 100 (Fig. 2).

### Assessment of paravalvular leakage

Experienced operators examined the amount of PVL in transthoracic echocardiography before discharge. Thereby, the PVL was visually graduated in none, trivial, mild, moderate and severe.

### Electrocardiogram monitoring

All patients remained on telemetric monitoring for a minimum of 48 h post-intervention. Furthermore, a twelve-lead electrocardiogram (ECG) was obtained in every patient pre-TAVI, daily in the initial two days after procedure and thereafter all two days until discharge. We added a 24-h Holter-ECG in patients with any conduction disturbances (CD) on twelve-lead-ECG or during monitoring. New CD after TAVI were defined as new onset of any kind of atrioventricular block or bundle branch block—if they persisted until discharge. A progress of an atrioventricular or a new bundle branch block was determined as CD in case of preexisting conduction disturbances. Patients with implanted pacemaker prior to TAVI were excluded from the subanalysis of predictors for CD after TAVI.

### Statistical analysis

We used SPSS software, Version 25.0 (IBM Corp., Armonk, NY, USA) and MedCalc, Version 19.4 (MedCalc Software Ltd., Ostend, Belgium) for the statistical analyzes. Categorical data are depicted as frequencies and percentages, continuous variables as mean with standard deviation or median with interquartile range. To test differences between two groups (e.g. between patients with and without CD) we used the χ2-test (for categorical variables), the Student`s t-test (for normal distributed continuous variables) or the Mann–Whitney-U Test (non-normal distributed continuous variables). Normal distribution was examined by the Kolmogorov–Smirnov-Test. To test the linear correlation between the implantation depth or annular/THV eccentricity and PVL we used the Spearman´s rank correlation. We used univariate and multivariate logistic regression models to assess possible predictors for new CD, a lower prosthesis position or LT. The multivariate models examined variables with a P-value < 0.1 in univariate analysis. A P value of < 0.05 was defined as statistically significant in all tests.

## Results

During the study period 118 patients with an implanted Evolut R THV received a post-TAVI CTA. The image quality of two post-TAVI CTAs was too poor to perform fusion imaging. Ten patients were excluded due to a valve-in-valve procedure and two patients received a surgical revision caused by a THV dislocation.

The mean age of the study cohort of 104 patients (66.3% female) was 82.2 ± 5.2 years with a mean logistic Euroscore of 15.1 ± 11.3%. Preexisting CD were documented in 48 patients (46.2%), five patients had a permanent pacemaker before TAVI.

The mean implantation depth of the THV in the whole cohort was 4.3 ± 3.0 mm below the annulus plane. There were significant differences in implantation depth when analyzing individual cusps separately with the right and left coronary cusp showing lower positions compared to the non-coronary cusp (4.9 ± 2.8 mm and 4.9 ± 3.4 mm vs. 3.1 ± 3.5 mm, P < 0.001).

All baseline, procedural and prosthesis-related characteristics are presented in Table 1.

**Table 1:** Baseline, procedural and prosthesis-related characteristics of the entire study population

		All patients (n = 104)
Age	(years)	82.2 ± 5.2
Female		69 (66.3)
BMI	(kg/m^2^)	27.8 ± 4.9
Logistic Euroscore	(%)	15.1 ± 11.3
Preexisting conduction disturbances	Pacemaker	5 (4.8)
	Total Conduction disturbances	48 (46.2)
	Atrioventricular block degree I (AV I)	14 (13.5)
	Atrioventricular block degree III (AV III)	2 (1.9)
	Left bundle branch block (LBBB)	17 (16.3)
	Right bundle branch block	4 (3.8)
	AV I + LBBB	5 (4.8)
	Bifascicular block	5 (4.8)
	Sick sinus syndrome	1 (1.0)
Atrial fibrillation		29 (27.9)
Aortic valve area	(cm^2^)	0.72 ± 0.22
Aortic valve type	Tricuspid	98 (94.2)
	Bicuspid	6 (5.8)
Annulus diameter	(mm)	23.1 ± 2.3
Annulus eccentricity (CTA)		1.3 ± 0.1
Grade of calcification of the device landing zone	total	4.2 ± 1.1
	Left coronary cusp	1.4 ± 0.5
	Right coronary cusp	1.3 ± 0.5
	Non-coronary cusp	1.5 ± 0.5
Grade of calcification of the LVOT	total	0.6 [0;1.0]
	Left coronary cusp	0.3 [0;0.7]
	Right coronary cusp	0 [0;0]
	Non-coronary cusp	0 [0;0.3]
Ejection fraction pre-interventional	(%)	50.6 ± 10.3
Access route	Transfemoral	103 (99.0)
	Trans-subcalvian	1 (1.0)
Prosthesis size	23 mm	5 (4.8)
	26 mm	46 (44.2)
	29 mm	42 (40.4)
	34 mm	11 (10.6)
Postdilatation		56 (53.8)
THV expansion stent center	(%)	90.0 ± 9.4
THV expansion LVOT end	(%)	62.9 ± 12.1
THV tilt	(°)	6.5 ± 3.9
Implantation depth below annulus (mm)	Mean	4.3 ± 3.0
	Left coronary cusp	4.9 ± 2.8
	Right coronary cusp	4.9 ± 3.4
	Non-coronary cusp	3.1 ± 3.5
Paravalvular leakage	None	34 (32.7)
	Trivial	25 (24.0)
	Mild	40 (38.5)
	Moderate	5 (4.8)
	Severe	0 (0)

Values are mean ± standard deviation, median [interquartile range] or n (%)

*AV* atrioventricular block degree, *BMI* body mass index, *CD* conduction disturbances, *CTA* computed tomography angiography, *LBBB* left bundle branch block, *LVOT* left ventricular outflow tract, *THV* transcatheter heart valve

## New conduction disturbances

Ninety-nine pacemaker naïve patients were monitored for CD post-TAVI. New onset CD developed in 54 (54.5%) of these patients.

Among the patients without preexisting CD 10 exhibited a left bundle branch block (LBBB) or a first and third degree atrioventricular block (each six patients). Of the patients with preexisting CD each four patients with first degree atrioventricular block developed a third degree atrioventricular block or a new LBBB (Table 2).

**Table 2:** Characterization of the new conduction disturbances (CD) after TAVI (n = 54) in patients with and without pre-existing CD

Pre-existing CD		New CD after TAVI	
AV I	8	AV III	4
		LBBB	4
LBBB	8	AV I	3
		AV II Mobitz type II	1
		AV III	1
		Progressive LBBB	3
AV I + LBBB	2	Progressive AV I	1
		Progressive AV I and LBBB	1
RBBB	1	AV III	1
Incomplete RBBB	1	LBBB	1
Bifascicular Block	3	AV I	1
		AV III	2

New CD after TAVI	
AV I	6
AV II Mobitz type II	1
AV III	6
LBBB	10
AV I + LBBBB	4
Incomplete LBBB	3
Sinuatrial block	1

Values are n (%)

*AV* atrioventricular block, *CD* conduction disturbances, *LBBB* left bundle branch block, *RBBB* right bundle branch block

Patients of both groups were comparable for baseline and procedural characteristics (Table 3). Patients with new CD revealed a significant lower mean stent position (5.2 ± 2.4 mm vs. 3.1 ± 3.3 mm, P < 0.001). This was also observed in the subanalysis of all three cusps (P < 0.001 for LCC and RCC, P = 0.004 for NCC). However, the grade of calcification of the device-landing zone was similar between the two groups—both in total and adjacent to the LCC, RCC or NCC (P = 0.438, P = 0.993, P = 0.650 and P = 0.140, respectively). Twenty-five patients (25.3%) received a permanent pacemaker implantation after TAVI.

**Table 3:** Baseline, procedural and prosthesis-related characteristics for pacemaker naïve patients (n = 99) with and without new conduction disturbances (CD) as well as for patients with a lower and higher prosthesis position

		Pacemaker naïve patients (n = 99)			THV position (n = 104)		
		With new CD	Without new CD	P-Value	Lower position (n = 66)	Higher position (n = 38)	P-Value
		(n = 54)	(n = 45)				
Age	(years)	82.9 ± 5.2	81.1 ± 4.8	0.084	82.3 ± 5.2	82.0 ± 5.1	0.735
Female		35 (64.8)	32 (71.1)	0.505	38 (57.6)	31 (81.6)	0.013
BMI	(kg/m^2^)	28.1 ± 4.8	27.5 ± 5.3	0.585	27.8 ± 4.8	27.9 ± 5.2	0.945
Logistic Euroscore	(%)	13.6 ± 9.6	16.1 ± 12.9	0.263	14.3 ± 10.0	16.5 ± 13.3	0.337
Previous pacemaker	Pacemaker	n.a	n.a	n.a	3 (4.5)	2 (5.3)	0.869
Previous CD		23 (48.1)	20 (44.4)	0.853	33 (50.0)	15 (39.5)	0.762
Aortic valve area	(cm^2^)	0.72 ± 0.23	0.74 ± 0.20	0.730	0.74 ± 0.24	0.70 ± 0.18	0.472
Aortic valve type				0.538			0.481
	Tricuspid	50 (92.6)	43 (95.6)		63 (95.5)	35 (92.1)	
	Bicuspid	4 (7.4)	2 (4.4)		3 (4.5)	3 (7.9)	
Annulus diameter	(mm)	23.1 ± 2.2	23.1 ± 2.4	0.658	23.6 ± 2.2	22.3 ± 2.1	0.002
Annulus eccentricity (CTA)		1.3 ± 0.2	1.3 ± 0.1	0.106	1.3 ± 0.2	1.3 ± 0.1	0.578
Grade of calcification of the device landing zone	total	4.3 ± 0.9	4.1 ± 1.3	0.438	4.4 ± 1.1	4.0 ± 1.2	0.123
	Left coronary cusp	1.4 ± 0.5	1.4 ± 0.5	0.993	1.5 ± 0.5	1.3 ± 0.5	0.060
	Right coronary cusp	1.3 ± 0.4	1.3 ± 0.5	0.650	1.3 ± 0.5	1.3 ± 0.5	0.576
	Non-coronary cusp	1.6 ± 0.5	1.4 ± 0.5	0.140	1.5 ± 0.5	1.4 ± 0.5	0.297
Grade of calcification of the LVOT	total	0.5 [0;1.1]	0.8 [0;1.1]	0.808	0.5 [0;1.1]	0.8 [0;1.1]	0.282
	Left coronary cusp	0.3 [0;0.8]	0.3 [0;0.5]	0.839	0.3 [0;0.8]	0.3 [0;0.5]	0.878
	Right coronary cusp	0 [0;0]	0 [0;0]	0.605	0 [0;0]	0 [0;0]	0.197
	Non-coronary cusp	0 [0;0.3]	0 [0;0.4]	0.702	0 [0;0.3]	0 [0;0.5]	0.558
Ejection fraction pre-interventional	(%)	52.6 ± 8.9	50.4 ± 9.6	0.239	50.4 ± 10.1	51.1 ± 10.9	0.750
Access route				0.359			0.446
	Transfemoral	53 (98.1)	45 (100)		65 (98.5)	38 (100)	
	Trans-subclavian	1 (1.9)	0 (0)		1 (1.5)	0 (0)	
Prosthesis size				0.708			0.006
	23 mm	3 (5.6)	1 (2.2)		4 (6.1)	1 (2.6)	
	26 mm	22 (40.7)	22 (48.9)		21 (31.8)	25 (65.8)	
	29 mm	24 (44.4)	17 (37.8)		34 (51.5)	8 (21.1)	
	34 mm	5 (9.3)	5 (11.1)		7 (10.6)	4 (10.5)	
Postdilatation		26 (48.1)	28 (62.2)	0.161	35 (53.0)	21 (55.3)	0.826
New CD after TAVI^a^		n.a	n.a	n.a	45 (68.2)	9 (23.7)	< 0.001
New PM after TAVI^a^		n.a	n.a	n.a	20 (31.7)	5 (13.9)	0.143
THV expansion stent center	(%)	89.5 ± 10.4	90.2 ± 7.6	0.307	89.4 ± 10.5	91.1 ± 7.1	0.082
THV expansion LVOT end	(%)	64.0 ± 11.4	60.5 ± 10.8	0.160	67.3 ± 11.8	55.1 ± 8.0	< 0.001
THV tilt	(°)	6.6 ± 3.7	6.5 ± 4.2	0.894	6.3 ± 3.9	6.9 ± 3.8	0.646
Implantation depth below annulus (mm)	Mean	5.2 ± 2.4	3.1 ± 3.3	< 0.001	n.a	n.a	n.a
	Left coronary cusp	5.8 ± 2.3	3.7 ± 2.8	< 0.001	n.a	n.a	n.a
	Right coronary cusp	5.9 ± 2.6	3.5 ± 3.8	< 0.001	n.a	n.a	n.a
	Non-coronary cusp	3.9 ± 2.8	2.0 ± 3.8	0.004	n.a	n.a	n.a
Paravalvular leakage				0.632			0.814
	None	16 (29.6)	16 (35.6)		20 (30.3)	14 (36.8)	
	Trivial	15 (27.8)	9 (20.0)		16 (24.2)	9 (23.7)	
	Mild	20 (37.0)	19 (42.2)		26 (39.4)	14 (36.8)	
	Moderate	3 (5.6)	1 (2.2)		4 (6.1)	1 (2.6)	
	Severe	0 (0)	0 (0)		0 (0)	0 (0)	

Values are mean ± standard deviation or n (%)

*CD* conduction disturbances, *CTA* computed tomography angiography, *LVOT* left ventricular outflow tract, *THV* transcatheter heart valve

^a^Percent value based on patients without previous permanent pacemakers

We included the age, logistic Euroscore, preexisting CD, total grade of calcification of the device-landing zone, prosthesis size, postdilatation, THV expansion of the stent center and the LVOT end as well as the mean implantation depth below annulus in the logistic regression models. After multivariate adjustment only the mean implantation depth was identified as predictor for new CD after TAVI (odds ratio [CI] 1.312[1.119–1.539], P = 0.001) (Table 4).

**Table 4:** Univariate and multivariate logistic regression model analysis of predictors of new conduction disturbances after TAVI

	Univariate		Multivariate	
	P-Value	Odds ratio [95% CI]	P-Value	Odds ratio [95% CI]
Age	0.087	1.075 [0.990–1.167]	0.126	1.071[0.981–1.170]
Logistic Euroscore	0.268	0.979 [0.944–1.016]		Not available
Preexisting CD	0.853	0.927 [0.418–2.060]		Not available
Total grade of device landing zone calcification	0.420	1.158 [0.811–1.655]		Not available
Prosthesis size	0.927	0.993 [0.854–1.155]		Not available
Postdilatation	0.163	0.564 [0.252–1.261]		Not available
THV expansion stent center	0.727	0.463 [0.006–35.202]		Not available
THV expansion LVOT end	0.128	17.761 [0.436–723.457]		Not available
Mean implantation depth below annulus	0.001	1.317 [1.124–1.543]	0.001	1.312[1.119–1.539]

*CD* conduction disturbances, *LVOT* left ventricular outflow tract, *THV* transcatheter heart valve

## Lower implantation depth

In the receiver operating characteristic curve analysis, a mean implantation depth of ≥ 4 mm demonstrated the best cut-off for occurrence of new CD post-TAVI (Sensitivity 83.3%, Specificity 60.0%). Therefore, we defined a lower implantation depth as at least 4 mm of the prosthesis below (ventricular) the native annulus plane. THVs were found in 66 patients (63.5%) in a lower and in 38 patients (36.5%) in a higher position. Using this cut off patients with a lower THV position developed more new CD after TAVI (68.2 vs. 23.7%, P < 0.001) (Table 3). Patients with a lower implantation depth were less often women (57.6 vs. 81.6%, P = 0.013), had a larger annulus diameter (23.6 ± 2.2 vs. 22.3 ± 2.1 mm, P = 0.002) and we found a significant difference for implanted valve sizes (P = 0.006). There was a trend for a higher calcification grade of the left coronary cusp (1.5 ± 0.5 vs. 1.3 ± 0.5 mm, P = 0.060).

Additionally, a lower prosthesis position is associated with a larger THV expansion at the LVOT end (67.3 ± 11.8 vs. 55.1 ± 8.0%, P < 0.001).

### Predictors for a lower THV position

We included gender, annulus diameter, prosthesis size and the calcification grade of the left coronary cusp in the logistic regression models. After multivariate adjustment, none of the variables were significantly predictive for a lower THV position (Data not shown).

### Paravalvular leakage

PVL was classified as none in 34 (32.7%), trivial in 25 (24.0%), mild in 40 (38.5%) and moderate in 5 (4.8%) patients (shown in Table 1). A lower THV position had no influence on the grade of PVL as shown by the Spearman´s rank correlation between the mean implantation depth and PVL (r = 0.052, P = 0.598, data not shown). Furthermore, neither the eccentricity of the native annular plane (r = 0.020, P = 0.841) nor of the deposited prosthesis on this height (r = 0.113, P = 0.255) were predictive for PVL. However, the grade of calcification has an influence on the grade of PVL (r = 0.298, P = 0.002).

## Discussion

In this comprehensive analysis using 3D fusion imaging of pre-and post-TAVI CTA we investigated the positioning of self-expandable THVs. In patients with TAVI using the Evolut R device a lower THV positioning is a predictor for new conduction disturbances. However, we could not identify any factors that predispose to low positioning. Implantation depth was not associated with the grade of PVL.

### New conduction disturbances after TAVI

After TAVI using SEV, 54.5% of the patients developed new CD, 25.3% received a permanent pacemaker implantation. These complications have potential effects on long-term outcomes as well as enormous influence on health economics [12]. Therefore, the identification of potential predictors for conduction disturbances is crucial.

In this study, a lower mean implantation depth was the only predictor for new CD using fusion imaging for a reliable 3-D depiction of the THV within the native annulus region. This results are in line with preexisting data for various valve types, where a deep prosthesis position in the LVOT, though only assessed in fluoroscopy or post-TAVI CTA, is a risk factor for CD [4, 5]. A mean implantation depth of ≥ 4 mm demonstrated the best cut-off for occurrence of new CD post-TAVI in our study. This value, measured by 3D fusion imaging, is in line with the results of the ADVANCE II trial, in which a prosthesis position shallower than 4 mm in the LVOT was associated with less permanent pacemaker implantations [13]. However, in ADVANCE II, the 2D angiographic depiction of the annulus is limited by its two-dimensionality and may be potentially misleading due to the complex geometry of the aortic annulus [7]. The manufacturer recommended a final implantation position with 3 to 5 mm of the prosthesis length below annular plane.

In contrast to previous studies investigating BEV, the calcification burden of the device landing zone had no influence on the occurrence of new CD [7, 14]. One might assume that through the self-expandable mechanism calcifications spots were pushed less into the surrounding tissue. Therefore, the resulting tissue damage of the conduction system in close proximity to the subaortic region and membranous septum seems to be lower.

### Low prosthesis position

Based on fusion imaging of pre- and post-TAVI CTA none of the structural or procedural related criteria revealed to be predictive for a low prosthesis position in SEV. In the light of a correlation of lower implantation depth and the occurrence of CD, a higher deposition of the prosthesis should be considered in all patients with pre-existing CD to avoid new CD. A former study of our group determined a reduced calcium burden within the cusp region as the sole independent predictor for a low position in patients with BEV [11].

### Paravalvular leakage

The geometric specialty of the Evolut R is a highest radial force at the LVOT edge of the prosthesis and a continuously decreasing diameter towards the stent center. With respect to this, a recently published study identified the implantation depth as predictor for moderate or severe PVL [15]. Our data showed no influence of the implantation depth or the eccentricity of the annular plane (native or the prosthesis on this height) on PVL. This could be explained by the fact that the mean implantation depth in our study was higher and more following the manufactures recommendation than in the above study (4.3 ± 3.0 mm vs. 6.2 ± 2.9 mm). According to previous publications, a higher calcification grade of the device landing zone is associated with a more pronounced PVL [16, 17].

In summary, based on our results of the correlation between the implantation depth a high THV deposition might be desirable to avoid pacemaker implantations. However, high positioning carries the risk of complete THV dislocation into the ascending aorta. Therefore, the implantation depth should be aimed at the lower range of the manufacturer recommendation with around 3 mm prosthesis length below the annular plane.

## Limitations

The limited sample size of our cohort limits the power to identify minor predictors for the occurrence of new CD or a lower prosthesis position. Especially the prevalence of right bundle branch block is low, what is a known preditor of worsening post TAVI conduction disturbances. While a lower mean implantation depth was a predictor for new CD, there was only a trend towards a higher PM implantation rate in these patients. A larger patient cohort might help to clarify the clinical significance of these CD. Moreover, our study was limited to patients with one specific SEV type. It remains speculative, if these results are transferable to other SEV types.

## Conclusion

In patients with TAVI using the Evolut R SEV, a lower THV positioning (≥ 4 mm length below annulus) was a predictor for new conduction disturbances. In contrast, implantation depth was not associated with the extent of PVL.

## Clinical perspectives

In the light of a further expansion of TAVI-procedure, an increased knowledge about the underlying mechanisms of possible complications is crucial for any specific valve design. Our study suggests that an implantation depth of ≥ 4 mm of the Evolut R leads to significantly more new CD after TAVI, whereas a higher position is not associated with more PVL. With respect to this, a deposition of the prosthesis above this value should be considered. Structural characteristics of the annular region showed no independent influence on implantation depth.

## Abbreviations

BEV: Balloon-expandable valves
CD: Conduction disturbances
CTA: Computed tomography angiography
ECG: Electrocardiogram
LVOT: Left ventricular outflow tract
PVL: Paravalvular leakage
SEV: Self-expanding valves
TAVI: Transcatheter aortic valve implantation
THV: Transcatheter heart valve
